# Optimal Volume of Moderate-to-Vigorous Physical Activity Postconcussion in Children and Adolescents

**DOI:** 10.1001/jamanetworkopen.2023.56458

**Published:** 2024-02-16

**Authors:** Andrée-Anne Ledoux, Veronik Sicard, Vid Bijelić, Nick Barrowman, Michael M. Borghese, Nicholas Kuzik, Mark S. Tremblay, Keith Owen Yeates, Adrienne L. Davis, Gurinder Sangha, Nick Reed, Roger Leonard Zemek

**Affiliations:** 1 Children’s Hospital of Eastern Ontario Research Institute, Ottawa, Ontario, Canada; 2 Department of Cellular and Molecular Medicine, University of Ottawa, Ottawa, Ontario, Canada; 3 Environmental Health Science and Research Bureau, Health Canada, Ottawa, Ontario, Canada; 4 Department of Pediatrics, Children’s Hospital of Eastern, Ontario, University of Ottawa, Ottawa, Ontario, Canada; 5 Department of Psychology, University of Calgary, Calgary, Alberta, Canada; 6 Alberta Children’s Hospital Research Institute and Hotchkiss Brain Institute, University of Calgary, Calgary, Alberta, Canada; 7 Department of Pediatrics, Hospital for Sick Children, Toronto, Ontario, Canada; 8 Department of Pediatrics, Children’s Hospital London Health Sciences Centre, Western University, London, Ontario, Canada; 9 Department of Occupational Science and Occupational Therapy, University of Toronto, Toronto, Ontario, Canada

## Abstract

**Importance:**

Determining the optimal volume of early moderate-to-vigorous-intensity physical activity (MVPA) after concussion and its association with subsequent symptom burden is important for early postinjury management recommendations.

**Objectives:**

To investigate the association between cumulative MVPA (cMVPA) over 2 weeks and subsequent symptom burden at 1 week, 2 weeks, and 4 weeks postinjury in children and examine the association between cMVPA and odds of persisting symptoms after concussion (PSAC) at 2 weeks and 4 weeks postinjury.

**Design, Setting, and Participants:**

This multicenter cohort study used data from a randomized clinical trial that was conducted from March 2017 to December 2019 at 3 Canadian pediatric emergency departments in participants aged 10.00 to 17.99 years with acute concussion of less than 48 hours. Data were analyzed from July 2022 to December 2023.

**Exposure:**

cMVPA postinjury was measured with accelerometers worn on the waist for 24 hours per day for 13 days postinjury, with measurements deemed valid if participants had 4 or more days of accelerometer data and 3 or fewer consecutive days of missing data. cMVPA at 1 week and 2 weeks postinjury was defined as cMVPA for 7 days and 13 days postinjury, respectively. Multiple imputations were carried out on missing MVPA days.

**Main Outcomes and measures:**

Self-reported postconcussion symptom burden at 1 week, 2 weeks, and 4 weeks postinjury using the Health and Behavior Inventory (HBI). PSAC was defined as reliable change on the HBI. A linear mixed-effect model was used for symptom burden at 1 week, 2 weeks, and 4 weeks postinjury with a time × cMVPA interaction. Logistic regressions assessed the association between cMVPA and PSAC. All models were adjusted for prognostically important variables.

**Results:**

In this study, 267 of 456 children (119 [44.6%] female; median [IQR] age, 12.9 [11.5 to 14.4] years) were included in the analysis. Participants with greater cMVPA had significantly lower HBI scores at 1 week (75th percentile [258.5 minutes] vs 25th percentile [90.0 minutes]; difference, −5.45 [95% CI, −7.67 to −3.24]) and 2 weeks postinjury (75th percentile [565.0 minutes] vs 25th percentile [237.0 minutes]; difference, −2.85 [95% CI, −4.74 to −0.97]) but not at 4 weeks postinjury (75th percentile [565.0 minutes] vs 25th percentile [237.0 minutes]; difference, −1.24 [95% CI, −3.13 to 0.64]) (*P* = .20). Symptom burden was not lower beyond the 75th percentile for cMVPA at 1 week or 2 weeks postinjury (1 week, 259 minutes; 2 weeks, 565 minutes) of cMVPA. The odds ratio for the association between 75th and 25th percentile of cMVPA and PSAC was 0.48 (95% CI, 0.24 to 0.94) at 2 weeks.

**Conclusions and Relevance:**

In children and adolescents with acute concussion, 259 minutes of cMVPA during the first week postinjury and 565 minutes of cMVPA during the second week postinjury were associated with lower symptom burden at 1 week and 2 weeks postinjury. At 2 weeks postinjury, higher cMVPA volume was associated with 48% reduced odds of PSAC compared with lower cMVPA volume.

## Key Points

**Question:**

What is the optimal volume of early moderate-to-vigorous physical activity (MVPA) postconcussion and its association with symptom burden?

**Findings:**

In this cohort study including 267 children and youths MVPA volumes up to 259 minutes in the first week and up to 565 minutes throughout the first 2 weeks were associated with lower symptom burden at week 1 and week 2.

**Meaning:**

These findings suggest that MVPA reduced symptoms up to a certain threshold for children and youths but appeared to offer no further benefit in symptom reduction beyond that point.

## Teaser

This cohort study investigates the association between cumulative moderate-to-vigorous-intensity physical activity throughout 2 weeks after a concussion and the symptom burden a 1 week, 2 weeks, and 4 weeks postinjury among youths in Canada.

## Introduction

Concussion poses a significant burden on population health and the economy. Globally, 50 to 60 million people are estimated to have a traumatic brain injury (TBI) each year, with 75% to 90% of hospital cases being TBIs or concussions.^1,2^ Approximately 30% to 35% of concussion cases will demonstrate persisting symptoms after concussion (PSAC).^3,4^ PSAC significantly impairs daily activities and quality of life,^5^ underscoring the urgent need to generate and refine best evidence management protocols. Early engagement in physical activity has been associated with reduced symptom burden at 2 weeks postinjury, faster recovery, and a lower risk of developing PSAC.^6,7,8,9,10,11,12,13,14,15,16,17,18,19,20,21,22^ However, the optimal volume of daily physical activity for achieving recovery in the general pediatric population remains unclear.^14^

The reference standard protocol for identifying the optimal intensity of physical activity postconcussion is the Buffalo Concussion Treadmill Test. This graded exercise test is designed to assess an individual’s tolerance for physical exertion and to guide the development of an individualized physical activity plan based on heart rate thresholds.^9,13^ However, many primary care clinicians do not have the equipment, personnel expertise, or time to routinely conduct this protocol in children and adolescents. Further, many children and adolescents do not have access to such specialized clinics.

Current research is limited and contradictory regarding the optimal volume and intensity of physical activity during the first weeks postinjury. In youths with acute sport-related concussion (SRC), engaging in higher volumes (approximately 72.4 minutes per day) of moderate to vigorous intensity physical activity (MVPA) was associated with longer recovery compared with lower volumes (approximately 36.9 minutes per day) during the first 3 days postconcussion.^10^ In contrast, adolescents with a SRC recruited within 10 days of injury who performed 20 minutes of individualized, subsymptom aerobic activity (based on heart rate threshold) per day for 4 weeks had reduced recovery time and risk of PSAC.^13^ In adolescents with SRC, a higher threshold of prescribed exercise volume (more than 160 minutes per day) was associated with being asymptomatic at 1 month.^7^ Moreover, adolescents with SRC who engaged in more than 30 minutes per day of MVPA for 7 days reported symptom resolution sooner compared with those who recorded less.^11^ Further, those completing 3 exercise sessions and 135 minutes per week of physical activity were medically cleared to return to play faster (less than 28 days) than those who did minimal exercise (more than 28 days).^8^ Studies that have investigated the optimal volume of physical activity have primarily focused on individuals with PSAC,^23,24,25,26^ used small sample sizes,^7,8,10,11,13^ only included sport-related populations,^7,8,9,10,11,13^ or lacked objective measurements to assess the intensity of physical activity performed by patients.^7,9^

The lack of prospective studies with rigorous methods on stepwise physical activity guidelines, specifically for the general pediatric population with acute concussion, has made it challenging for clinicians to implement standardized recommendations. This study had 3 goals: (1) to investigate the association between cumulative MVPA (cMVPA) volume and total subsequent symptom burden at 1 week, 2 weeks, and 4 weeks postinjury; (2) to investigate the association between cMVPA volume and the risk of developing PSAC at 2 weeks and 4 weeks postinjury; and (3) to explore the association with cMVPA and subsequent cognitive and somatic symptoms at 1 week, 2 week, and 4 weeks postinjury. We hypothesized that increased cMVPA would be associated with decreased symptom burden at 1 week, 2 weeks, and 4 weeks postinjury, and decreased the odds of PSAC at 2 weeks and 4 weeks postinjury.

## Methods

This cohort study was a planned secondary analysis of data collected from the Pediatric Concussion Assessment of Rest and Exertion study (PedCARE, NCT02893969),^27^ a multicenter clinical trial. Participants were recruited from March 2017 to December 2019 in 3 emergency departments (EDs) affiliated with the Pediatric Emergency Research Canada (PERC) network. The PedCARE study was approved by the ethics committees of all 3 participating institutions. All participants provided written informed assent or consent and parents provided consent. This study follows the Strengthening the Reporting of Observational Studies in Epidemiology (STROBE) reporting guideline.

### Study Population

Youth aged 10.00 years to 17.99 years who sustained a concussion within 48 hours of presenting to the ED were included in this study. Concussions were defined based on the Zurich and Berlin consensus statement for SRC.^28,29^ An adapted version of the US Centers for Disease Control and Prevention tiered framework was used to confirm concussion in participants.^30^ Those with either 1 highest level of certainty signs (eg, retrograde or anterograde amnesia; loss of consciousness) or 2 higher level of certainty signs or symptoms (eg, nausea or vomiting; headache; dizziness) immediately or within 1 hour of injury were included. Exclusion criteria were as follows: Glasgow Coma Scale score of 13 or less; abnormality on brain imaging if completed; neurosurgical intervention, intubation, or intensive care unit admission; multisystem injury requiring hospitalization; severe preexisting neurological developmental delay resulting in communication difficulties; intoxication; absence of trauma history as primary event; previously enrolled; insurmountable language barrier; or inability to complete follow-ups.

### Study Protocol

The study protocol and primary analyses have been published.^6,27^ Participants completed questionnaires collecting demographics, injury characteristics, personal health, and mental health history. Balance was assessed using the Balance Error Scoring System (BESS).^31^

The Health and Behavior Inventory (HBI)^32,33^ and retrospective HBI were used to measure the participant’s preinjury and postinjury symptom profile and severity. The HBI is a 20-item (score range 0 to 60, higher scores indicate increased number of symptoms or more severe symptoms) valid and reliable symptom rating scale recommended as a National Institutes of Health core common data element for concussion.^34,35^ The HBI queries symptoms in somatic (9 items; range 0 to 27) and cognitive (11 items; range 0 to 33) domains.^36^ Parents completed the retrospective HBI in the ED, and participants completed the HBI at 1 week, 2 weeks and 4 weeks postinjury via the Research Electronic Data Capture (REDCap)^37,38^ database or phone.

Next, participants were randomized to either the experimental (ie, start return-to-physical activity at 72 hours) or control group (ie, return to physical activity once asymptomatic). Participants received standardized instructions on return-to-learn and return-to-physical activity,^28,39,40,41^ concussion education, and were given an Actical accelerometer to provide valid and reliable estimates of physical activity.^42^ The accelerometer was worn on a belt around the waist at the right midaxillary line for 14 consecutive days (24 hours per day) post ED-visit, and collected data in 1 minute epochs (excluding aquatic activities).

### Outcome Measures

Symptom burden was defined as the sum score of the 20 items on the HBI. The cognitive and somatic symptom scores were defined as the sum score of 11 and 9 items, respectively.

PSAC was determined with reliable change *z* scores.^36^ The reliable change score compares the parent’s retrospective rating of preinjury total symptoms with the child’s rating of symptoms reported during the 2 week and 4 week follow-up, based on formulae derived from regression analyses in children with orthopedic injuries. PSAC was defined by a reliable change *z* score of 1.65 or more, which indicates a greater-than-expected increase in symptoms based on retrospective parent preinjury ratings.

Postinjury *z* score for total score = postinjury child total score – (6.352 + [0.476 × retrospective parent preinjury total score]) / 9.597

### Data Processing

Data processing procedures have been published elsewhere.^6^ Time spent in each movement intensity was classified according to established intensity cut-offs for the accelerometer: vigorous physical activity, 6500 or more counts per minute; moderate physical activity, between 6499 and 1500 counts per minute; light physical activity, between 1499 and 100 counts per minute^43^; and sedentary behaviors, less than 100 counts per minute.^44^

cMVPA at 1 week was defined as the sum of MVPA for days 1 to 7 after the ED visit. cMVPA at 2 weeks and 4 weeks was defined as the sum of MVPA for days 1 to 13 after the ED visit. cMVPA (for week 1 and week 2) was used for the analysis of 2 weeks and 4 weeks postinjury.

### Statistical Analysis

Based on Ledoux et al,^6^ no differences in mean MVPA were found between the experimental and control groups, and therefore they were combined in the current analysis. Data for MVPA per day were included if participants had at least 4 days of accelerometer data (with more than 8 hours per day of waking wear time) and 3 or fewer consecutive days of incomplete data.

We used multiple imputations using chained equations to account for missing estimator and outcome values.^44^ A estimated mean matching strategy was used to impute missing values from numeric variables (continuous or ordinal), and logistic regression was used for categorical variables. The imputation model included total HBI score, number of minutes of MVPA per day, and 56 additional variables selected from the PedCARE data set known to be potential confounders or predictors of symptom burden or PSAC (eTable 1 in Supplement 1). Finally, the imputation model included auxiliary variables associated with nonresponse, which were preselected using the algorithm of van Buuren et al.^45^

Modeling results using the multiple imputed data sets were combined into final estimates based on Rubin rules.^45^ The model estimates were assessed for the relative efficiency of imputation, and an efficiency of 95% or higher was sought.

A linear mixed-effects model was fitted with HBI score as dependent variable to assess the association between cMVPA in the previous week and symptom burden at 1 week, 2 weeks, and 4 weeks postinjury. Fixed effects were cMVPA, cMVPA at each follow-up time point, time, randomization group, age, sex, Predicting Persistent Postconcussive Problems in Pediatrics (5P) risk score,^4^ preinjury mental health and learning disability comorbidity (concatenated variable, including anxiety, depression, sleep disorder, other mental health disorder, learning disabilities, attention disorder, and other developmental disorder, coded as 1 or 0), and retrospective and ED HBI score. The random effect was participants. Contrasts were specified between the 75th vs 25th percentile to provide estimates of effect size associated with cMVPA.

 Two multivariable logistic regression models were computed to investigate the association of cMVPA in the previous week and PSAC odds at 2 weeks and 4 weeks postinjury. Fixed factors were cMVPA, age, sex, 5P risk score, mental health and learning disability comorbidity, and ED HBI.

Exploratory linear mixed-effects analyses were fitted with the scores as dependent variables to assess the association between cMVPA in the previous week and cognitive and somatic HBI scores at 1 week, 2 weeks, and 4 weeks postinjury. For both models, we used nonimputed data (ie, HBI cognitive and somatic scores). Participants were excluded only if they lacked HBI scores at all time points. Fixed and random factors were the same as analysis 1.

For all analyses, a restricted cubic spline (3 knots) was applied to cMVPA and age to allow for nonlinearity. cMVPA was treated as a time-varying variable. Nonlinearity for all models was assessed by reviewing locally estimated scatterplot smoothing (LOESS) curves and if necessary, the application of a restricted cubic spline to compare the model fit with a linear term was conducted. For all analyses, 2-sided *P* < .05 was considered statistically significant, and mice version 3.16.0 and R version 4.0.5 (R Project for Statistical Computing) were used.^46,47^

## Results

**Figure 1.**
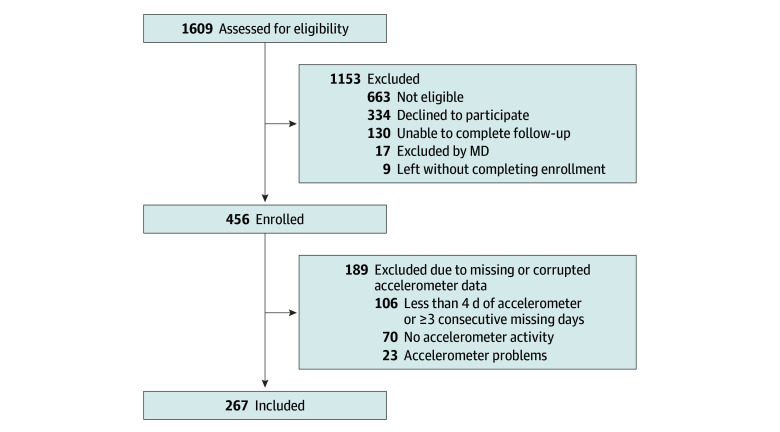
Flow Diagram MD indicates medical doctor.

In this study, 267 of 456 children from the larger trial sample (119 [44.6%] female; median [IQR] age, 12.9 [11.5-14.4] years) were included in the analysis (Figure 1); 189 (41.4%) were lost due to missingness in the activity monitor data. Personal and injury characteristics are presented in Table 1. Median (IQR) cMVPA was 146.0 (90.8-264.2) minutes at 1 week postinjury and 380.5 (235.8-589.5) minutes at 2 weeks postinjury. Median (IQR) HBI scores were 18.0 (10.0-29.0) at 1 week postinjury, 14.0 (5.0-23.0) at 2 weeks postinjury, and 10.0 (1.5-19.0) at 4 weeks postinjury (eTable 2 in Supplement 1).

**Table 1.** Personal Characteristics

Variable	Participants, No. (%)			
	Complete sample	Analytical sample	Missing, in analytical sample	Data without or insufficient accelerometer data
Total, No.	456	267	NA	189
Age, median (IQR), y	13.1 (11.5-14.8)	12.9 (11.5-14.4)	0	13.4 (11.8-15.5)
Sex				
Female	201 (44.1)	119 (44.6)	0	82 (43.4)
Male	255 (55.9)	148 (55.4)	0	107 (56.6)
5P clinical risk score, median (IQR)	7.0 (5.0-8.0)	7.0 (5.0-8.0)	1 (0.4)	7.0 (5.0-8.0)
Time from head injury to triage, median (IQR), h	3.0 (1.6-20.0)	3.0 (1.6-20.0)	3 (1.1)	3.1 (1.6-19.7)
Personal migraine history	25 (5.5)	11 (4.1)	0	14 (7.4)
Previous concussions	150 (32.9)	86 (32.2)	0	64 (33.9)
Previous number of concussions, median (IQR)	1.0 (1.0-2.0)	1.0 (1.0-2.0)	181 (67.8)	1.0 (1.0-2.0)
Symptom duration of prior concussion of 1 week or more	101 (22.1)	52 (19.5)	0	49 (25.9)
Parent-reported diagnostic history				
Learning disabilities	52 (15.5)	33 (14.9)	46 (17.2)	19 (16.5)
Attention-deficit hyperactivity disorder	48 (14.3)	33 (14.9)	46 (17.2)	15 (13.0)
Other developmental disorder	20 (6.0)	12 (5.4)	46 (17.2)	8 (7.0)
Anxiety	60 (17.9)	34 (15.4)	46 (17.2)	26 (22.6)
Depression	20 (6.0)	8 (3.6)	46 (17.2)	12 (10.4)
Sleep disorder	13 (3.9)	9 (4.1)	46 (17.2)	4 (3.5)
Other psychiatric disorder	7 (2.1)	4 (1.8)	46 (17.2)	3 (2.6)
Mechanism of injury			1 (0.4)	
Occupant in motor vehicle collision	6 (1.3)	4 (1.5)	0	2 (1.1)
Pedestrian struck by auto	1 (0.2)	1 (0.4)	0	0
Bike struck by auto	0	0	0	0
Bike collision or fall while riding	4 (0.9)	2 (0.8)	0	2 (1.1)
Fall from an elevation (including playground equipment)	14 (3.1)	9 (3.4)	0	5 (2.7)
Fall downstairs	8 (1.8)	3 (1.1)	0	5 (2.7)
Sport	238 (52.4)	144 (54.1)	0	94 (50.0)
Fall from standing, walking, or running	63 (13.9)	37 (13.9)	0	26 (13.8)
Ran into stationary object	24 (5.3)	16 (6.0)	0	8 (4.3)
Other mechanism	96 (21.1)	50 (18.8)	0	46 (24.5)
Loss of consciousness	81 (17.8)	48 (18.0)	0	33 (17.5)
Duration of loss of consciousness				
<1 min	60 (74.1)	37 (77.1)	0	23 (69.7)
1-5 min	18 (22.2)	9 (18.8)	0	9 (27.3)
>5 min	3 (3.7)	2 (4.2)	0	1 (3.0)
Assessed in the ED				
Seizure	4 (0.9)	2 (0.7)	0	2 (1.1)
Answers questions slowly	204 (44.7)	113 (42.3)	0	91 (48.1)
Headache	417 (91.4)	241 (90.3)	0	176 (93.1)
Sensitivity to noise	254 (55.7)	153 (57.3)	0	101 (53.4)
Fatigue	392 (86.0)	230 (86.1)	0	162 (85.7)
Balance error score tandem stance ≥4	51 (11.2)	38 (14.3)	1 (0.4)	13 (6.9)
Retrospective HBI, median (IQR)	13.0 (6.5-20.0)	12.5 (6.0-19.0)	1 (0.4)	14.0 (7.0-20.0)
HBI at ED, median (IQR)	23.0 (16.0-31.0)	21.0 (15.0-31.0)	1 (0.4)	25.0 (18.0-31.2)
cMVPA, median (IQR)				
cMVPA week 1	NA	146.0 (90.8-264.2)	99 (37.1)^a,b^	NA
cMVPA week 2	NA	380.5 (235.8-589.5)	147 (55.1)^a,c^	NA

Abbreviations: 5P, Predicting Persistent Postconcussive Problems in Pediatrics; cMVPA, cumulative moderate-to-vigorous-intensity physical activity; ED, emergency department; HBI, Health and Behavior Inventory; NA, not available.

^a^ Missing because of noncompliance or low wear time.

^b^ Missing at any day over the first week.

^c^ Missing at any day over the first 2 weeks.

### cMVPA and Recovery Trajectory

**Figure 2.**
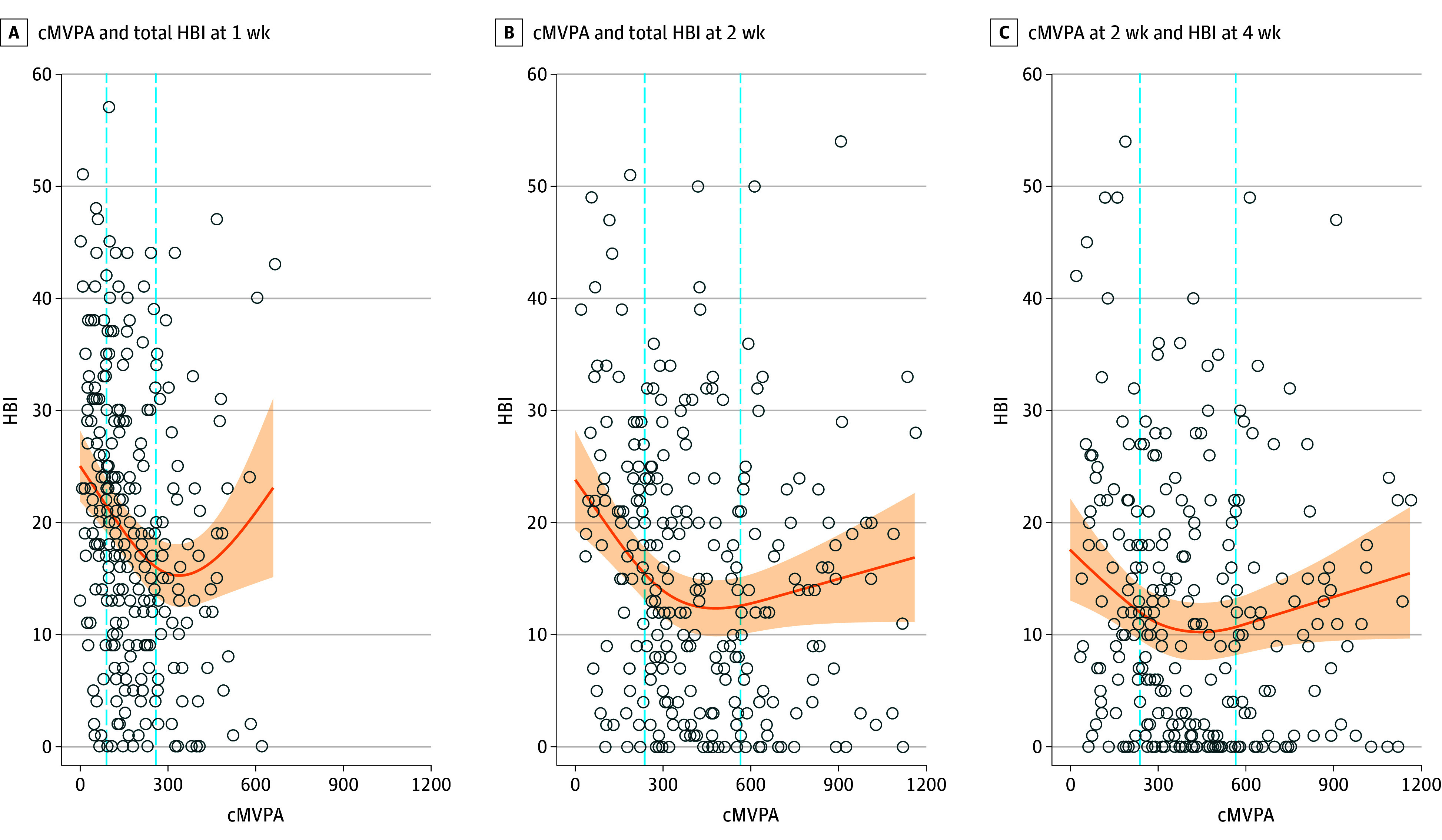
Cumulative Moderate-to-Vigorous-Intensity Physical Activity (cMVPA) and Total Health Behavior Inventory (HBI) Score Curves are adjusted for randomized treatment group, age, sex, Predicting Persistent Postconcussive Problems in Pediatrics risk score, mental health and learning disability comorbidity, retrospective HBI score, and emergency department HBI score. Circles represent imputed data of the first imputation. Dashed lines represent the 25th and 75th quantiles of cMVPA. Shaded areas represent the 95% CIs.

Multiple imputation was carried out and pooled model results showed relative efficiencies of 99% and higher for all variables. LOESS curves based on observed and imputed data for HBI vs cMVPA at 1 week, 2 weeks, and 4 weeks postinjury are shown in the eFigure in Supplement 1. Participants with greater cMVPA had significantly lower HBI scores at 1 week (75th percentile [258.5 minutes] vs 25th percentile [90.0 minutes]; difference, −5.45 [95% CI, −7.67 to −3.24]) and 2 weeks postinjury (75th percentile [565.0 minutes] vs 25th percentile [237.0 minutes]; difference, −2.85 [95% CI, −4.74 to −0.97]) but not at 4 weeks postinjury (75th percentile [565.0 minutes] vs 25th percentile [237.0 minutes]; difference, −1.24 [95% CI, −3.13 to 0.64]) (*P* = .20) (Figure 2). Symptom burden was not lower beyond the 75th percentile for cMVPA at 1 week or 2 weeks postinjury (1 week, 259 minutes; 2 weeks, 565 minutes) of cMVPA. Past these thresholds, our estimated model suggested either no improvement or an increase in symptoms. Nonlinearity was significant *P* < .001. cMVPA percentiles for 1 week, 2 weeks, and 4 weeks postinjury are presented in Table 2.

**Table 2.** Cumulative Moderate-to-Vigorous Physical Activity Percentiles at 1 Week and 2 Weeks

Week	10th percentile	25th percentile	Median	75th percentile	90th percentile
1	45.00	90.00	151.00	258.50	381.00
2^a^	125.00	237.00	366.00	565.00	797.20

^a^ The same percentiles were used to estimate the 4 week outcome.

### cMVPA and Odds of PSAC at 2 Weeks and 4 Weeks Postinjury

Additionally, 29 participants (10.9%) were reliably symptomatic at 2 weeks postinjury, and 19 participants (7.1%) were reliably symptomatic at 4 weeks postinjury. The *P* values for nonlinear fit were significant at 2 weeks postinjury (*P* <.001) and were not significant at 4 weeks postinjury. The effect size of cMVPA on PSAC was significant at 2 weeks postinjury (*P* = .001) but was not significant at 4 weeks postinjury. The estimated probability of PSAC at the 25th and 75th percentiles of cMVPA was 0.07 (95% CI, 0.03-0.14) vs 0.03 (95% CI, 0.01-0.09) at 2 weeks postinjury and 0.07 (95% CI, 0.03-0.14) vs 0.04 (95% CI, 0.02-0.12) at 4 weeks postinjury. When contrasting cMVPA at the 75th vs 25th percentile at 2 weeks and 4 weeks postinjury, the odds ratio for PSAC was 0.48 (95% CI, 0.24-0.94; *P* = .03) and 0.65 (95% CI, 0.30-1.37; *P* = .25), respectively.

### cMVPA and Cognitive and Somatic Recovery Trajectory

**Figure 3.**
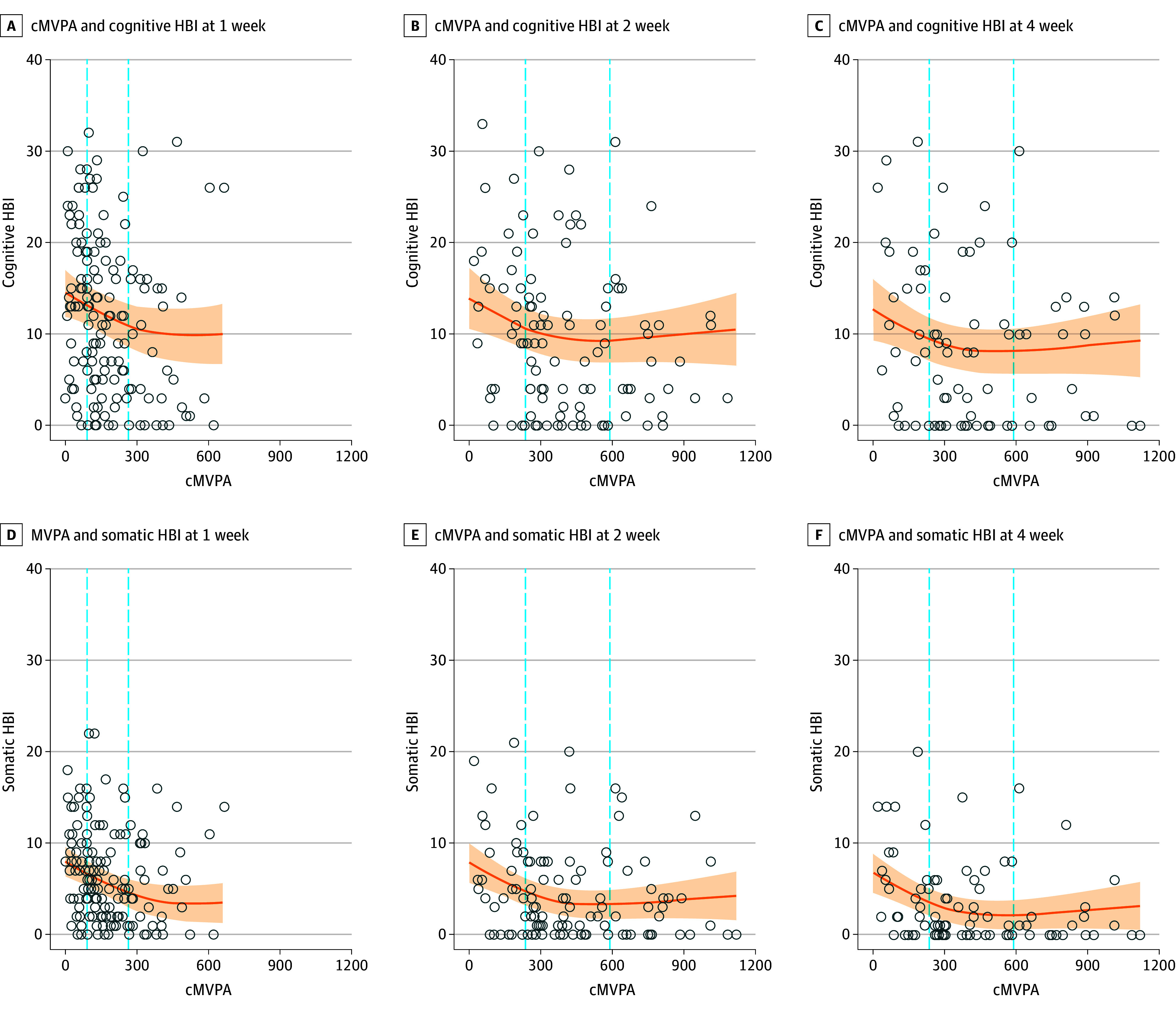
Cumulative Moderate-to-Vigorous-Intensity Physical Activity (cMVPA) and Health Behavior Inventory (HBI) Cognitive Score and Somatic Score Curves are adjusted for randomized treatment group, age, sex, 5P risk score, mental health and learning disability comorbidity, retrospective HBI score, and emergency department HBI score. Circles represent imputed data of the first imputation. Dashed lines represent the 25th and 75th quantiles of cMVPA. Shaded areas represent the 95% CIs.

Given that the interaction of time and cMVPA was not significant, it was removed from the model. Significant nonlinearity was found for both cognitive and somatic models. cMVPA was associated with reduced cognitive (F_2, 271.9_ = 4.23; *P* = .016) and somatic symptom burden (F_2, 277.8_ = 9.15; *P* < .001) (Figure 3). When comparing the weekly 25th and 75th percentiles of cMVPA for both symptom scores, the slope of the graph was significant for cognitive symptom burden at 1 week postinjury and for somatic symptom burden at 1 week, 2 weeks, and 4 weeks postinjury (eTable 3 in Supplement 1).

## Discussion

Among children and adolescents with acute concussions, engaging in higher volumes of MVPA within the first week postinjury (259 vs 90 minutes) or within the first 2 weeks postinjury (565 vs 237 minutes) was associated with lower symptom burden. Beyond these thresholds, no clear benefit in symptom burden was apparent. The cMVPA does not appear to have a greater impact on reducing somatic symptoms vs cognitive symptoms. Higher volumes of cMVPA at 2 weeks postinjury (565 vs 237 minutes) was associated with 48% lower odds of PSAC.

Nationally representative physical activity data from Statistics Canada indicate that the mean daily minutes of MVPA is 65 minutes for children aged 6 to 11 years and 52 minutes for adolescents aged 12 to 17 years, which is much higher than the levels observed in our study population.^48^ This is not surprising and may be attributed to the injury itself, because adolescents may not have fully returned to their regular activities and may engage in more light or sedentary behaviors after concussion, as demonstrated in our previous research.^49^ Interestingly, we found that the association between cMVPA and symptom burden appeared to plateau after the 259 minute in the first week and exceeding 375 minutes of cMVPA during the first week may have a negative impact on recovery. The pattern of our data seemed to be consistent with a Goldilocks effect (optimal range), with higher symptom burden at both very low levels and very high levels of MVPA, but this could not be confirmed due to wider confidence intervals beyond the higher thresholds. Our data suggested that cMVPA was associated with reduced symptoms within a threshold and was no longer beneficial past these thresholds. Further investigation with a larger sample size is required to confirm the suspected Goldilocks effect.

A small sample size study on subacute SRC found that more than 30 minutes per day of MVPA between 7 to 14 days postinjury was associated with decreased time to symptom resolution.^11^ Other groups have found increased physical activity to be associated with faster symptom resolution,^9^ full resolution of symptoms at 4 weeks,^7^ faster medical clearance to return-to-play,^8^ and decreased PSAC risk.^13^ Our findings aligned with these studies and suggested that an even higher threshold of 259 minutes of cMVPA within the first week is beneficial. Consistent with the existing research,^12,13,50^ our results indicated that individuals who engaged in a moderate volume of MVPA had significantly lower odds of experiencing PSAC. In a large prospective observational study, where physical activity was based on self-report questionnaire, those who returned to physical activity within 7 days of concussion had a 24.6% risk of PSAC at 4 weeks postinjury compared with a 43.5% risk in those who had not returned to physical activity.^12^ The risk of PSAC in those who resumed full contact physical activity was 14.5% compared with 43.5% in those who did not resume full-contact physical activity. A recent randomized clinical trial involving adolescents within 10 days of SRC reported a 48% lower risk of PSAC at 4 weeks among those assigned to a prescribed exercise program (20 minutes per day) compared with those assigned to a placebo stretching program.^13^ Further aligning with previous studies, we found increased cMVPA volume at 2 weeks was associated with 48% lower PSAC odds.

The present study has several strengths, including a relatively large sample and objective evaluation of cMVPA. Unlike studies that relied on questionnaires to assess the volume of, or adherence to, physical activity, our study used accelerometer-based measurement on a 24-hour basis. This research was conducted across 3 Canadian centers on a variety of concussion mechanisms, enhancing the generalizability and external validity of our results. Additionally, we used a standardized definition of PSAC. This standardized approach ensured consistency in identifying and categorizing individuals with PSAC, enhancing the reliability of our results and facilitating comparisons with other studies.^51^

We would like to emphasize the importance and benefits of gradually returning to routine activities, including physical activity, following a concussion. Several studies have highlighted the positive impacts of prescribed physical activity in managing symptoms. Engaging in physical activity can contribute to the overall well-being of children and adolescents during their recovery process and reduce the odds of PSAC. However, our data also suggested that exceeding 300 minutes in the first week (equivalent to more than 43 minutes of MVPA per day) had no effect on symptoms and might even result in increased symptoms. In this study, some participants who engaged in a high volume of MVPA had no symptom improvement, suggesting that they may not be adequately adjusting their activity levels in response to their symptoms. In contrast to individuals who were avoiding physical activity, those who were overexercising might require a different clinical approach. As outlined in concussion guidelines,^39,52^ patients should gradually resume physical activity as long as their symptoms remain tolerable. This gradual approach ensures a safe and effective return to physical activity while minimizing the risk of exacerbating symptoms.

### Limitations

This study has limitations. While the results provide valuable insights into the association between cMVPA and symptom burden, they do not establish a causal association and are potentially limited by reverse causation. The nature of the study design precludes establishing the directionality of the observed associations. Moreover, children and adolescents with worse symptoms or PSAC may have been less compliant at wearing the accelerometer or in MVPA participation, or conversely those less motivated to wear the accelerometer could differ in both activity levels and recovery. To mitigate this possibility, we adjusted for baseline symptom severity and 5P risk score. Future studies using RCT designs should investigate the association with cMVPA in the first 2 weeks postinjury and concussive symptom resolution.

The crude nature of the cMVPA metric provides no information on the temporality of accumulation during the designated time (1 week or 2 weeks postinjury). Furthermore, we have no information on cMVPA in weeks 3 and 4 postinjury, limiting our understanding of cMVPA variability in the past 2 weeks. Although our sample included participants with various mechanisms of injury, they were all recruited in the ED. This may introduce selection bias, as individuals who sought medical attention may differ from those who did not in terms of injury severity or mechanism of injury. Another limitation was the presence of missing activity monitor data. To address this issue, we used multiple imputation techniques to estimate missing values in participants with sufficient data. The imputation introduces some degree of uncertainty in the analysis, and the accuracy of the imputed values relies on the underlying assumptions made during the imputation process. However, when comparing the imputed data to complete case data, our results are consistent (eFigure in Supplement 1).

## Conclusions

In children and adolescents with acute concussion, 259 minutes of cMVPA during the first week postinjury and 565 minutes of cMVPA during the second week postinjury were associated with lower symptom burden. At 2 weeks postinjury, higher cMVPA volume compared with lower volume was associated with 48% reduced odds of PSAC.

## Notes

Supplement 1. **eFigure.** LOESS Curve of Unadjusted Observed and Imputed Distribution With 95% CI of Cumulative Moderate to Vigorous Physical Activity (cMVPA) and Health and Behavior Inventory (HBI) at 1, 2, and 4 Weeks **eTable 1.** Additional Variables Included in the Imputation Model **eTable 2.** HBI Scores Through Time **eTable 3.** Contrast for Exploratory Analysis

Supplement 2.
Data Sharing Statement
