# Supplementary material for: Optimal Volume of Moderate-to-Vigorous Physical Activity Postconcussion in Children and Adolescents
Source: JAMA Netw Open. 2024 Feb 16;7(2):e2356458. doi: 10.1001/jamanetworkopen.2023.56458 (PMC10873766; doi:10.1001/jamanetworkopen.2023.56458)
Supplement: Supplement 1. — eFigure. LOESS Curve of Unadjusted Observed and Imputed Distribution With 95% CI of Cumulative Moderate to Vigorous Physical Activity (cMVPA) and Health and Behavior Inventory (HBI) at 1, 2, and 4 Weeks eTable 1. Additional Variables Included in the Imputation Model eTable 2. HBI Scores Through Time eTable 3. Contrast for Exploratory Analysis [file jamanetwopen-e2356458-s001.pdf]

Supplemental Online Content

Ledoux AA, Sicard V, Bijelic V, et al. Optimal volume of moderate-to-vigorous physical activity postconcussion in children and adolescents. *JAMA Netw Open*. 2024;7(2):e2356458. doi:10.1001/jamanetworkopen.2023.56458

eTable 1. Additional Variables Included in the Imputation Model

eTable 2. HBI Scores Through Time

eTable 3. Contrast for Exploratory Analysis

eFigure. LOESS Curve of Unadjusted Observed and Imputed Distribution With 95% CI of Cumulative Moderate to Vigorous Phsyical Activity (cMVPA) and Health and Behavior Inventory (HBI) at 1, 2, and 4 Weeks

This supplemental material has been provided by the authors to give readers additional information about their work.

© 2024 Ledoux AA et al. *JAMA Network Open.*

eTable 1. Additional Variables Included in the Imputation Model

Variable	MinValue	MaxValue	ImputationMethod
symptom	0.00	2.00	2lonly.pmm
randomization	0.00	1.00	2lonly.pmm
site	0.00	2.00	2lonly.pmm
sex	0.00	1.00	2lonly.pmm
calc_age	10.01	17.86	2lonly.pmm
mechanism_injury	0.00	11.00	2lonly.pmm
Lost of consciousness	0.00	1.00	2lonly.pmm
seizure	0.00	1.00	2lonly.pmm
amnesia_before	0.00	1.00	2lonly.pmm
amnesia_hurt	0.00	1.00	2lonly.pmm
amnesia_after	0.00	1.00	2lonly.pmm
headache	0.00	1.00	2lonly.pmm
pain_scale_response	1.00	10.00	2lonly.pmm
pain_upset	0.00	4.00	2lonly.pmm
noise	0.00	1.00	2lonly.pmm
fatigue	0.00	1.00	2lonly.pmm
disorientation	0.00	1.00	2lonly.pmm
nauseous	0.00	1.00	2lonly.pmm
vomiting	0.00	1.00	2lonly.pmm
dizziness	0.00	1.00	2lonly.pmm
vision	0.00	2.00	2lonly.pmm
balance	0.00	1.00	2lonly.pmm
light	0.00	1.00	2lonly.pmm
ment_foggy	0.00	1.00	2lonly.pmm
slow_down	0.00	1.00	2lonly.pmm
concentrating	0.00	1.00	2lonly.pmm
remembering	0.00	1.00	2lonly.pmm
irritable	0.00	1.00	2lonly.pmm
sad	0.00	1.00	2lonly.pmm
emotional	0.00	1.00	2lonly.pmm
nervousness	0.00	1.00	2lonly.pmm
sleep_less	0.00	2.00	2lonly.pmm
sleep_more	0.00	2.00	2lonly.pmm
trouble_sleep	0.00	2.00	2lonly.pmm

© 2024 Ledoux AA et al. *JAMA Network Open.*

Variable	MinValue	MaxValue	ImputationMethod
additional_injuries	0.00	1.00	2lonly.pmm
prev_concussion	0.00	1.00	2lonly.pmm
past_headaches	0.00	1.00	2lonly.pmm
dx_migraines	0.00	1.00	2lonly.pmm
answer_slowly	0.00	1.00	2lonly.pmm
bess_able	0.00	2.00	2lonly.pmm
learning_disabilities	0.00	1.00	2lonly.pmm
attention_disorder	0.00	1.00	2lonly.pmm
other_dev_disorder	0.00	1.00	2lonly.pmm
dx_anxiety	0.00	1.00	2lonly.pmm
dx_depression	0.00	1.00	2lonly.pmm
dx_sleep_disorder	0.00	1.00	2lonly.pmm
dx_other_psych	0.00	1.00	2lonly.pmm
annual_income	0.00	5.00	2lonly.pmm
mother_education	0.00	9.00	2lonly.pmm
father_education	0.00	9.00	2lonly.pmm
bess_double_errors	0.00	10.00	2lonly.pmm
bess_tandem_errors	0.00	10.00	2lonly.pmm
activities_60_mins	0.00	3.00	2lonly.pmm
activities_breath	0.00	4.00	2lonly.pmm
activities_watch_tv	0.00	5.00	2lonly.pmm
activities_computer	0.00	5.00	2lonly.pmm

Notes. activities_60_mins = Health Canada Physical Activity Survey, over a typical week prior to the concussion, number of days the child is physically active for more than 60 min per day (none, 1 day, 2-3 days, 4 days or more); activities_breath = Health Canada Physical Activity Survey, over a typical week prior to the concussion, hours a week the child take part in physical activity that makes them out of breath or warmer than usual outside of school while participating in lessons or league or team sports (never, less than 1 per week, 2 to 3 hours per week, 4 to 6 hours per week, 7 or more hours per week); activities_watch_tv = Health Canada Physical Activity Survey, over a typical week prior to the concussion, on average, how many hours a day does the child watch TV or videos or play video games (doesn't watch TV or videos or play video games, less than 1 hours per day, 1 to 2 hours a day, 3 to 4 hours a day, 5 to 6 hours a day, 7 or more hours a day); activities_computer = activities_watch_tv = Health Canada Physical Activity Survey, over a typical week prior to the concussion, on average, how many hours a day does the child spend on a computer/cellphone (working, playing games, emailing, chatting, surfing the Internet; doesn't use a computer, less than 1 hour per day, 1 to 2 hours a day, 3 to 4 hours a day, 5 to 6 hours a day, 7 or more hours a day); annual_income = household annual income ($25,000 or less; $25,0001-$50,000; $50,001-$75,000; $75,001-$100,000; more than $100,000; does not wish to answer); answer_slowly = question to the parent, since the injury, does the child answer questions slowly? (yes or no); bess = Balance Error Scoring System; bess_able = whether or not the patient is able to complete the BESS; calc_age = age in years with decimals; concentrating = difficulty concentrating; dx = diagnosis; father_education = highest level of education of the father/legal guardian (high school not completed; secondary [high school] diploma or equivalency certificate; registered apprenticeship or other trades certificate or diploma; university degree – bachelor degree; master's degree; doctorate; other; NA [e.g., single parent, deceased, etc.]; does not wish to answer); loc = loss of consciousness; mechanism_injury = circumstances of

© 2024 Ledoux AA et al. *JAMA Network Open.*

injury (occupant in motor vehicle collision, motorcycle accident, motorized recreation vehicle accident, pedestrian struck by auto, bike struck by auto, bike collision or fall while riding, fall from an elevation, fall down stairs, sport, fall from standing/walking/running, ran into stationary object, other mechanism); ment_foggy = feeling mentally foggy; mother_education = highest level of education of the father/legal guardian (high school not completed; secondary [high school] diploma or equivalency certificate; registered apprenticeship or other trades certificate or diploma; university degree – bachelor degree; master's degree; doctorate; other; NA [e.g., single parent, deceased, etc.]; does not wish to answer); pain_scale response = headache pain scale at ED visit, rating from 0 to 10; pain_upset = headache pain scale at ED visit, rating from 0 (does not bother or upset you at all) to 4 (bothers or upsets you very much); prev_concussion = previous concussion (yes or no); randomization = randomized control trial randomization group (experimental or control); remembering; difficulty remembering; slow_down = feeling slowed down; symptom = average daily log symptom rating (0=a lot, 1=a little, 2=a lot) during the study. Signs (e.g., loc, amnesia, seizure) and symptoms (e.g., headache, fatigue, dizziness, etc) refer to those at those at time of injury. Premorbid developmental or mental health diagnoses are binary variables (yes or no).

eTable 2. HBI Scores Through Time

**Variable**	Analytic sample (Actical sample), *Median (IQR)*	Data without or insufficient Actical data, *Median (IQR)*	Missing information (Analytic sample), *frequency* (%)
Retrospective HBI	12.5 (6.0, 19.0)	14.0 (7.0, 20.0)	1 (0.4)
HBI at ED	21.0 (15.0, 31.0)	25.0 (18.0, 31.2)	1 (0.4)
HBI at Week 1	18.0 (10.0, 29.0)	20.0 (13.0, 32.0)	27 (10.1)
HBI at Week 2	14.0 (5.0, 23.0)	15.0 (7.5, 23.0)	25 (9.4)
HBI at Week 4	10.0 (1.5, 19.0)	14.0 (3.5, 21.0)	84 (31.5)

Notes. HBI = Health and Behaviour Inventory; IQR = Interquartile range; ED = Emergency Department.

eTable 3. Contrast for Exploratory Analysis

**Week**	**Contrast cMVPA p25-p75**		**Estimate (SE) [95%CI]**	***p*.value**
$1_{\text{cog. HBI}}$	90.75	235.75	-2.20 (0.78) [-3.72, -0.67]	0.005
$2_{\text{cog. HBI}}$	235.75	589.50	-1.35 (0.82) [-2.95, 0.25]	0.10
$4_{\text{cog. HBI}}$	235.75	589.50	0.25 (0.82) [0.82, 0.25]	0.10
$1_{\text{som. HBI}}$	90.75	264.25	-2.15 (0.51) [-3.15, -1.15]	< 0.001
$2_{\text{som. HBI}}$	235.75	589.50	-1.39 (0.53) [-2.43, -0.35]	0.009
$4_{\text{som. HBI}}$	235.75	589.50	-1.39 (0.53) [-2.43, 0.35]	0.009

Notes. 95%CI = 95% Confidence Interval; cMVPA = Cumulative Moderate-to-Vigorous Physical Activity; Cog. HBI = Cognitive score on the Health Behaviour Inventory (HBI); p25 = 25th percentile; p75 = 75th percentile; Som. HBI = Somatic score on the HBI.

© 2024 Ledoux AA et al. *JAMA Network Open.*

eFigure 1. LOESS Curve of Unadjusted Observed and Imputed Distribution With 95% CI of Cumulative Moderate to Vigorous Physical Activity (cMVPA) and Health and Behaviour Inventory (Hbi) at 1, 2, and 4 Weeks

Observed data

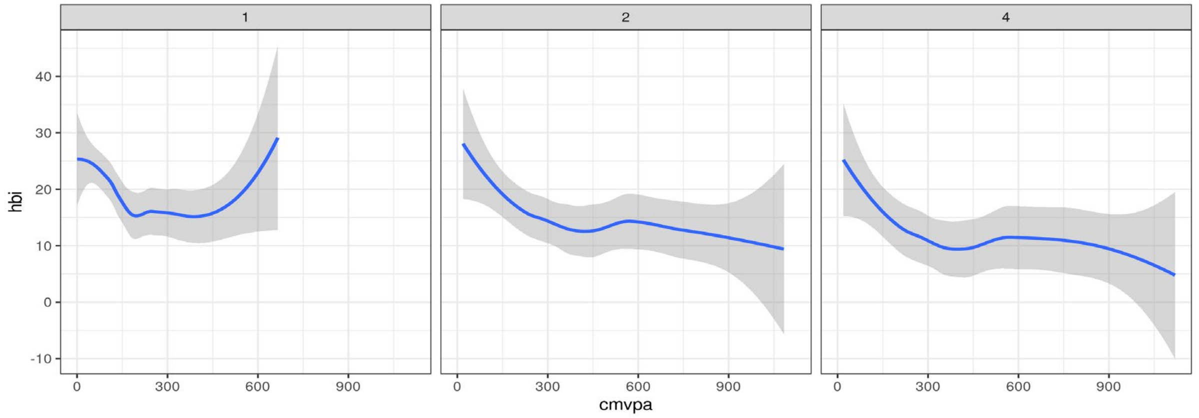

Imputed data

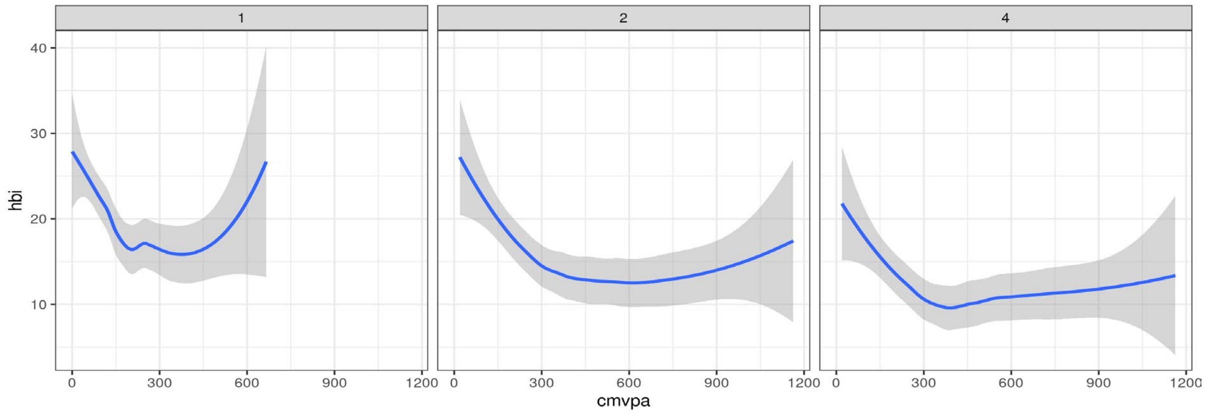

© 2024 Ledoux AA et al. *JAMA Network Open.*
