# Supplementary material for: Optimal Volume of Moderate-to-Vigorous Physical Activity Postconcussion in Children and Adolescents
Source: JAMA Netw Open. 2024 Feb 16;7(2):e2356458. doi: 10.1001/jamanetworkopen.2023.56458 (PMC10873766; doi:10.1001/jamanetworkopen.2023.56458)
Supplement: Supplement 2. — Data Sharing Statement [file jamanetwopen-e2356458-s002.pdf]

Data Sharing Statement

Data
Data available: No

Additional Information
Explanation for why data not available: The data that support the findings of this study are available on reasonable request from the corresponding author with accordance to ethical and legal regulations regarding the sharing of information as to prevent compromising the privacy of research participants.
